# Mobile health may improve evaluation of lower urinary tract symptoms

**DOI:** 10.1590/S1677-5538.IBJU.2021.0211.1

**Published:** 2021-10-01

**Authors:** Cristiano M. Gomes, Julyana K. M. Moromizato, Lucia S. Ribeiro

**Affiliations:** 1 Faculdade de Medicina da Universidade de São Paulo Departamento de Cirurgia Divisão de Urologia São PauloSP Brasil Divisão de Urologia do Departamento de Cirurgia da Faculdade de Medicina da Universidade de São Paulo – FMUSP, São Paulo, SP, Brasil

## COMMENT

The evaluation of patients with lower urinary tract symptoms (LUTS) is primarily based on clinical history and physical examination. Measuring the frequency and severity of LUTS adds important information for the characterization and management of lower urinary tract disorders (1, 2). Bladder diaries and uroflowmetry may be invaluable for symptom characterization. The recording of volume and time of each void by the patient is referred to as a frequency volume chart (FVC). Inclusion of information like fluid intake, use of pads, incontinence episodes or symptom severity is termed a bladder diary (1).

The FVC provides data on total voided volume, day-time and night-time voiding frequency, nocturnal urinary volume and individual voided volumes. The maximum voided volume (MVV) is an important parameter of the voiding diary as it corresponds to the functional bladder capacity. It may be important to improve our understanding of bladder sensation, overactive bladder symptoms, polyuria and overflow incontinence (3). It may be used to clinically phenotype patients and help counseling regarding fluid intake and timed voiding (3). It may also assist in monitoring patient´s response to treatment (2). The amount of information to be included and the duration of a bladder diary is variable, but typically should take from three to seven days (4).

In this study, based on the perception that patients often have a greater MVV during office uroflowmetry than that seen in the bladder diary, the authors compared these two non-invasive methods by which MVV can be determined. They used a database of over seven hundred patients evaluated for LUTS who completed a 24-hour bladder diary independently using a smartphone application. They found that there is a difference between the two measurement tools, and that the maximum voided volume recorded in a bladder diary (BD-MVV) is usually greater than that obtained at the time of uroflow (Q-MVV). They suggested that for a more reliable assessment of MVV in men and women, both Q-MVV and BD-MVV should be assessed and that the larger of the two values is a more reliable assessment of MVV (5).

It is important to highlight the use of a mobile app for the completion of the voiding diary in this study.Mobile health (mHealth) is an attractive and expanding tendency within LUTS care, both from the viewpoint of urologists and also by health systems and for research (6, 7). Typically, mHealth is based on a smartphone app that may help in the evaluation, monitoring and/or treatment of a health condition. mHealth has been used in urology for prostate cancer, urinary stones, LUTS, urinary incontinence and urinary tract infections and its use has gained importance with the COVID-19 pandemic (8). In the urinary stone field, apps may prevent forgetting a double-J catheter (9). For prostate cancer, apps may help physicians to stage patients and calculate disease risk and they may help patients to track symptoms and medications and connect them to care providers (10).

We were not able to analyze the app used in this study because it is proprietary and not open to download. Many apps are available for urinary incontinence/LUTS and anyone can download some of these apps to their phones. Typically, apps in this ﬁeld allow for symptom evaluation and monitoring or guide patients on how and when to do pelvic floor exercises and lifestyle adaptations (11). For doctors, apps may offer the possibility of improving the understanding of patient symptoms and might help selecting individualized diagnostic and treatment strategies. In addition, they may monitor patient’s progress and improve adherence to the treatment plan. Patients can have access to their progress and may become active participants in their own health care.

In spite of the interest in mhealth for UI/LUTS, a recent study evaluated the available apps in this field in Brazil and found that currently available tools are of poor quality (11). Most have unattractive layouts, low-resolution graphics and low-quality information, from questionable sources. Authors cautioned that there is a lack of evidence-based scientiﬁc information associated with these apps and they credited that to the fact that most apps were developed for commercial purposes, suggesting the need to promote better partnership between industry and academic institutions to improve the quality of healthcare apps.

Similarly, a systematic review of apps for UI/LUTS in English language found that many of the available apps are of questionable quality, mostly not based on credible sources and/or scientific evidence (8). Authors emphasize that future app development should focus on enhancing overall quality while including evidence-based content; in addition, the inclusion of features that might help increasing adherence to treatment would be valuable.

We still have many obstacles before the widespread use of mhealth technologies becomes a reality, especially with the elderly population. The use of mobile apps may be limited by their extension and complexity of their commands. Ideally, they should be as short and straightforward as possible, enabling easy and rapid completion, which may help expand their usage and improve their accuracy (8, 11, 12).

Improving the evidence-based scientiﬁc information attesting the efficacy of mHealth will be important to stimulate more practitioners and patients to adopt it. The present study represents one more step in this direction. If proven to be effective, apps providing evaluation and self-management of LUTS may increase access to care and reduce costs with treatment.
